# A novel biosensor for measuring plasmin activity

**DOI:** 10.1016/j.rpth.2026.106629

**Published:** 2026-05-02

**Authors:** Ying Dai, Paul Y. Kim, Stefan Heitmeier, Jeffrey I. Weitz, Peter L. Gross

**Affiliations:** 1 Thrombosis and Atherosclerosis Research Institute, Hamilton and Departments of Medicine and Medical Sciences, McMaster University, Hamilton, Canada; 2 Department of Medicine, University Health Network, University of Toronto, Toronto, Canada; 3 Department of Cardiovascular Research, Bayer AG, Research and Development Pharmaceuticals, Wuppertal, Germany

**Keywords:** antifibrinolytic agents, biosensing techniques, fibrin clot lysis time, fibrinolysin, protein interaction domains and motifs

## Abstract

**Background:**

Plasmin facilitates fibrinolysis by catalyzing the degradation of fibrin clots. Reliable methods to assess fibrinolysis by measuring plasmin generation are needed in the preclinical and clinical settings.

**Objectives:**

This study aimed to design, synthesize, and evaluate a fluorescence resonance energy quenching–based plasmin sensor (FPS) protein to monitor plasmin generation.

**Methods:**

The hydrolysis of FPS was monitored by loss of fluorescence resonance energy quenching to evaluate efficiency and specificity. Plasma-based plasmin generation assays were used to evaluate fibrinolytic cofactors and inhibitors.

**Results:**

Compared with a peptide plasmin sensor, FPS demonstrated higher efficiency for plasmin and was not cleaved by plasmin inhibited by α_2_-macroglobulin. FPS interacts with plasmin’s kringle domain 5. FPS can measure plasmin generation in clotting plasma, which was increased by an inhibitory antibody against α_2_-antiplasmin and attenuated by aprotinin, tranexamic acid, and fibrin absence. Maximal plasmin generation occurs after peak clot formation. Furthermore, when antiplasmin is inhibited, the presence of plasminogen activator inhibitor-1 can be measured. Unlike peptide sensors, FPS does not inhibit clot lysis. Lastly, plasmin generation measured using FPS is relatively consistent in plasma from healthy volunteers, with only a 2-fold variation in peak plasmin, peak time, and endogenous plasmin potential.

**Conclusion:**

FPS may improve the evaluation of fibrinolysis in preclinical and clinical settings, thereby enhancing our understanding of fibrinolytic disorders.

## Essentials

• Peptide plasmin sensors measure plasmin complexed with α_2_-macroglobulin (α_2_-M) and inhibit fibrinolysis.
• We produced a more efficient protein-based sensor that does not measure plasmin entrapped by α_2_-M.
• This sensor interacts with kringle 5 of plasmin, revealing novel interactions.
• By adding a blocking antibody to α_2_-antiplasmin, this sensor can detect nanomolar levels of plasmin.

## 1 Introduction

Hemostasis depends on a dynamic balance between clot formation and lysis [1]. Vascular damage results in activation of platelets and coagulation factors, which lead to thrombin generation, the mediator of clot formation [2]. Fibrinolysis degrades the hemostatic clot to restore blood flow in the vessel [3].

Plasmin is the main effector of fibrinolysis through its ability to cleave fibrin clots [1]. Excess plasmin activity leads to bleeding, while insufficient plasmin activity leads to thrombosis [4]. Plasminogen is activated to plasmin by tissue-type plasminogen activator (tPA) on fibrin clots and by urokinase-type plasminogen activator (uPA) on cell surfaces [5]. tPA and uPA are inhibited by plasminogen activator inhibitor (PAI)-1 [2]. In contrast, plasmin is inhibited covalently by α_2_-antiplasmin (AP) and sterically by α_2_-macroglobulin (α_2_-M) [2].

Typically, plasmin generation in citrated clotting plasma is assessed by monitoring the hydrolysis of peptide detectors such as Boc-Glu-Lys-Lys-AMC or Cbz-Phe-Arg-rhodamine-morpholino, in response to added tPA [1,6]. These methods yield plasmin generation curves from which outputs such as lag time, velocity rate, peak height, peak time and endogenous plasmin potential (EPP), and fibrin lysis time can be calculated [1,6]. Although these peptide sensors are generally specific for plasmin relative to other proteases, they measure both free plasmin and plasmin complexed with α_2_-M [1,7]. In addition, they have low catalytic efficiency for plasmin [[7], [8], [9]].

Previously, we developed a protein-based thrombin sensor that has high efficiency and specificity for thrombin relative to small peptide thrombin substrates and is resistant to hydrolysis by α_2_-M–bound thrombin [10] These characteristics arise from the ability of protein-based sensors to accommodate long enzyme recognition sequences, potentially form exosite interactions with the protease, and to be sensitive to inhibition by large inhibitors that entrap the protease [10]. Using the same backbone as our previously developed thrombin sensor, we have engineered a fluorescence resonance energy quenching (FREQ)-based plasmin sensor (FPS) protein. In this study, we characterized FPS as a novel, highly sensitive method of measuring plasmin generation that is resistant to α_2_-M–bound plasmin, enabling simultaneous measurement of plasmin generation and clot lysis.

## 2 Methods

### 2.1 A model of FPS

FPS consists of a plasmin recognition site (GVYKSRSL), flanked on both sides by antiparallel β sheets and the FREQ pair of mAmetrine and tTomato [11,12]. FPS has 2 connected N-terminal 6-histidine tags and a C-terminal calmodulin-binding protein tag (Supplementary Figure 1).

### 2.2 FPS expression and purification

The FPS sequence in the pESRTA vector was transformed into Rosetta (DE3) pLysS Competent *Esherichia coli* (Novagen) according to the manufacturer’s protocol. Bacteria were plated onto Luria-Bertani (LB) agar plates containing 100 μg/mL ampicillin (Bioshop) and grown overnight at 37 °C. FPS colonies were inoculated into 3 mL of LB broth supplemented with 100 μg/mL ampicillin and 10 μg/mL chloramphenicol (Sigma) and incubated overnight at 30 °C. The cultures were then diluted into 400 mL of LB broth (also containing ampicillin and chloramphenicol), grown to an OD600 of 0.5 at 37 °C while shaking at 250 rpm, induced with 0.1 mM isopropyl β-d-1-thiogalactopyranoside (Sigma) at 15 °C, and harvested after overnight induction.

The cells were pelleted, resuspended in buffer with an added protease inhibitor cocktail, lysed by sonication, and column purified as previously described [10]. After elution from the nickel column, purified FPS was dialyzed in Tris-buffered saline (TBS) containing 20 mM Tris-HCl, 50 mM KCl, 2 mM CaCl_2_, and 600 mM Trehalose (Sigma) as a stabilizer, at pH 8.0, before use. The purity of FPS was assessed by SDS-PAGE analysis under reducing conditions followed by Coomassie Brilliant Blue G-250 (Bio-Rad) staining using standard protocols.

### 2.3 Measurement of FPS and Boc-Glu-Lys-Lys-AMC hydolysis

Hydrolysis of FPS was monitored in wells of Nunc 96-well clear flat-bottom polystyrene plates (Thermo Fisher Scientific), measuring changes in 526 nm emission after 406 nm excitation fluorescence. Hydrolysis of Boc-Glu-Lys-Lys-AMC (Bachem) was monitored in a solid-bottom 96-well Assay Plate (VWR), measuring changes in 460 nm emission after 360 nm excitation fluorescence. Both were assessed at 37 °C using a SpectraMax M3 or M5.

### 2.4 Mini-plasminogen and micro-plasminogen activation

To obtain mini-plasmin or micro-plasmin, 5 μM of mini-plasminogen or micro-plasminogen (made in-house) was incubated with 100 nM uPA (Calbiochem) in TBS at 23 °C [13]. An aliquot containing 50 nM of mini-plasminogen or micro-plasminogen was removed, and conversion to mini-plasmin or micro-plasmin was determined by monitoring the hydrolysis of 400 μM S-2251 (Chromogenix) at 405 nm.

### 2.5 Michaelis–Menten kinetics parameters of FPS and Boc-Glu-Lys-Lys-AMC

FPS diluted in TBS containing 600 mM Trehalose to concentrations up to 10 μM, or Boc-Glu-Lys-Lys-AMC at concentrations up to 5 mM, was incubated with 5 nM plasmin, 5 and 10 nM mini-plasmin for FPS and Boc-Glu-Lys-Lys-AMC, respectively, or 50 and 30 nM micro-plasmin for FPS and Boc-Glu-Lys-Lys-AMC, respectively. Initial rates of hydrolysis were obtained and converted to micromolars cleaved using a standard curve. Initial rates were analyzed using a Michaelis–Menten model in GraphPad Prism to obtain *K*_m_ and *k*_cat_.

### 2.6 Activation of plasminogen derivatives by tPA in buffer measured using FPS and Boc-Glu-Lys-Lys-AMC

Activation of Glu-plasminogen and mini-plasminogen (both at 50 nM) were measured using of FPS (1 μM) [13]. These reagents were incubated with 1 μM fibrinogen and 0 to 64 μM tranexamic acid (TXA; Sigma) in TBS containing 0.004% Tween 20 (Bio-Rad) and 0.005% Prionex (Pentapharm). Reactions were initiated with 10 nM thrombin (Enzyme Research Laboratories) and 0.25 nM tPA (Roche). Cleavage rates were quantified to derive the area under the curve, peak height, and peak time for each assay.

In separate experiments, 1 μM FPS or 1 mM Boc-Glu-Lys-Lys-AMC was also used to measure activation of Glu-plasminogen (50 nM) in the same condition using 0.1, 0.05, and 0.025 nM tPA and 5 nM thrombin. For these experiments, the rates of substrate hydrolysis used to derive the area under the curve, peak height, and peak time were converted to nanomolars of plasmin generated using standard curves produced by cleaving the same concentration of FPS and Boc-Glu-Lys-Lys-AMC with purified plasmin (Prolytix) in the same buffer containing 1 μM fibrinogen along with 5 nM thrombin.

### 2.7 Evaluating the effects of TXA on cleavage of FPS by mini-plasmin and plasmin

FPS (1 μM) was cleaved using 10 nM mini-plasmin or plasmin in the presence and absence of 20 μM TXA in TBS containing 0.004% Tween 20, and initial rates were obtained for these conditions.

### 2.8 Quantifying the specificity of the substrates for free plasmin in comparison with other proteases

To determine the specificity of FPS and Boc-Glu-Lys-Lys-AMC for plasmin, 1 μM of FPS or 1 mM of Boc-Glu-Lys-Lys-AMC was incubated with 10 nM plasmin; 100 nM thrombin; activated protein C; factor [F]IXa, FXa, and kallikrein; 50 and 100 nM FXIa for FPS and Boc-Glu-Lys-Lys-AMC, respectively (Enzyme Research Laboratories); and 100 nM tPA or uPA. The rate of substrate cleavage by α_2_-M–bound plasmin was determined using 5 nM plasmin with or without preincubation with 100 nM α_2_-M (Sigma) for 1 hour at ambient temperature. The substrates, α_2_-M and enzymes were all diluted in TBS with 0.004% Tween 20 prior to the experiments.

### 2.9 Plasmin generation in plasma measured using FPS and Boc-Glu-Lys-Lys-AMC

The study protocol for collection of blood samples was approved by the Hamilton Integrated Research Ethics Board in Ontario, Canada. Informed consent was obtained from healthy volunteers. Blood was collected and prepared from healthy volunteers to obtain pooled platelet-poor plasma (PPP) as previously described [14].

Plasmin generation assays were performed where 0.25 to 1 μM FPS was added to 1/3.4 diluted pooled PPP in the presence of 5 nM thrombin and 7.4 nM tPA; in some experiments 0.25, 0.1, 0.05, or 0.025 nM tPA were used instead. For the experiments performed using 0.1, 0.05, and 0.025 nM tPA, PPP was purchased from Precision Biologic, and these experiments were performed in the presence of 0.5 μM of an antibody against AP (AP AB; a gift from Bayer). As a negative control, no tPA was added for 1 of the experiments. For these procedures, rates were quantified using the first derivative of changes in 526 nm emission to output plasmin generation curves. These curves were then converted to nanomolar of plasmin generated using standard curves obtained from cleaving 1 μM of FPS with purified plasmin (Prolytix) in AP-deficient plasma in the presence of 5 nM thrombin. The resulting plasmin generation curves were then used to derive peak plasmin, peak time, and EPP (Supplementary File Data Processing) [15].

To evaluate plasmin generation initiated using 0.1, 0.05, and 0.025 nM tPA in the presence of 0.5 μM AP AB, 1 mM Boc-Glu-Lys-Lys-AMC was also used. Similar to those with FPS, rates of cleavage of Boc-Glu-Lys-Lys-AMC were analyzed using the first derivative of changes in 460 nM emission to obtain plasmin generation curves. These curves were also converted to nanomolars of plasmin generated by cleaving 1 mM of Boc-Glu-Lys-Lys-AMC with purified plasmin (Prolytix) in AP-deficient plasma in the presence of 5 nM thrombin to obtain the same plasmin generation parameters [15].

Plasmin generation assays (1 μM of the FPS) were also performed where various percentages of AP-deficient plasma (Affinity Biologicals) were mixed with pooled PPP. Plasmin generation was also measured in pooled PPP in the presence or absence of 0.5 μM AP AB or 1 μM aprotinin (Sigma) [16]. Plasmin generation assays were repeated in the absence or presence of (1) TXA at varying concentrations up to 318 μM or (2) 1 mM of the fibrin polymerization inhibitor glycyl-L-prolyl-L-arginyl-L-proline (GPRP; Bachem), with or without 0.5 μM AP AB. Plasmin generation assays were also performed in defibrinated plasma (Affinity Biologicals) or PAI-1–deficient plasma (Affinity Biologicals) in the absence or presence of 0.5 μM AP AB.

### 2.10 Clot lysis assays

To perform clot lysis assays, 5 nM thrombin and 7.4 nM tPA were pipetted to opposite sides of a well, and then up to 1 μM FPS and 1/3.4 diluted PPP were added to a final volume of 100 μL. All reagents were diluted in TBS, and the final reaction also contained 0.004% Tween 20 and 0.005% Prionex. These experiments were performed in the SpectraMax M3 or M5 at 37°C using Nunc 96-well clear flat-bottom polystyrene plates, where absorbance was measured at 405 nm to quantify clot lysis, which was determined using the time from half maximal clotting to half maximal lysis. FREQ analysis to measure plasmin generation and 405 nm absorbance were measured simultaneously using the workflow setting. In other experiments, 0.1 and 0.025 nM tPA was used instead in the presence of 0.5 μM AP AB. For these experiments, the addition of 1 μM FPS and 1 mM Boc-Glu-Lys-Lys-AMC was compared with when TBS was added to the samples. Experiments were also performed using 0.1 nM tPA in the presence of 0.5 μM AP AB where the addition of 1 mM D-Ala-Phe-Lys-ANSNH-iC_4_H_9_·2HBr (SN-5; Prolytix) was compared with the addition of TBS. In separate experiments, plasmin generation and clot lysis assays were also performed using individual, not pooled, plasma samples from 13 healthy volunteers, aged 27 to 56 years.

### 2.11 Statistical analysis

Unpaired *t*-tests were used to determine the differences between 2 groups. One-way analysis of variance (anova), followed by Tukey multiple comparisons test, assessed differences across groups. Two-way anova, followed by Tukey multiple comparisons test, was used to assess differences across groups when there was >1 factor affecting the groups. Pearson correlation coefficient was used to calculate the correlation between 2 group parameters. *F*-tests were used to determine whether slopes are significantly different from zero. All statistical analyses were completed using GraphPad Prism, and a *P* < .05 was considered statistically significant.

## 3 Results

### 3.1 Plasmin cleavage changes the FREQ efficiency of FPS

SDS-PAGE analysis revealed a single FPS band of 68 kDa (Supplementary Figure 2A). Cleavage of FPS by plasmin leads to a 156% increase in fluorescence emission at 526 nm, while fluorescence emission at 581 nm decreased by 59%, corresponding to a loss of FREQ (Supplementary Figure 2B).

### 3.2 Cleavage of the FPS by plasmin is largely dependent on interactions between the substrate and the fifth kringle domain of plasmin

The catalytic efficiency of FPS hydrolysis by plasmin was 9-fold higher than that of Boc-Glu-Lys-Lys-AMC, mainly reflecting a lower *K*_m_ (Table 1). The catalytic efficiency (*k*_cat_/*K*_m_) of FPS hydrolysis by miniplasmin, comprising the catalytic domain and kringle domain 5, was similar to that of intact plasmin (Table 1). However, mini-plasmin was only 13% as efficient for Boc-Glu-Lys-Lys-AMC compared with plasmin (Table 1). Micro-plasmin, which lacks all kringle domains, demonstrated only 7% catalytic efficiency for FPS compared with plasmin (Table 1), where the effect is largely through *k*_cat_, and not *K*_m_. This contrasts with Boc-Glu-Lys-Lys-AMC, where the catalytic efficiency of micro-plasmin is 49% of that of plasmin (Table 1). These data suggest that, unlike Boc-Glu-Lys-Lys-AMC, FPS engages with plasmin specifically via its kringle domain 5.

**Table 1:** Kinetic parameters of FPS and Boc-Glu-Lys-Lys-AMC cleavage by plasmin and variants lacking Kringle domains.

Enzyme	Substrate	*K*_m_ (μM)	*k*_cat_ (s^−1^)	*k*_cat_/*K*_m_ (s^−1^ μM^−1^)	Relative *k*_cat_/*K*_m_ (plasmin)
Plasmin (5 nM)	FPS	1.4 ± 0.39	0.24 ± 0.023	0.18 ± 0.052	1.0 ± 0.42
Mini-plasmin (5 nM)	FPS	2.5 ± 1.0	0.44 ± 0.074	0.18 ± 0.080	1.0 ± 0.54
Micro-plasmin (50 nM)	FPS	1.7 ± 0.86	0.022 ± 0.0040	0.013 ± 0.0067	0.073 ± 0.044
Plasmin (5 nM)	Boc-EKK-AMC	240 ± 140	5.1 ± 0.76	0.021 ± 0.012	1.0 ± 0.83
Mini-plasmin (10 nM)	Boc-EKK-AMC	380 ± 160	1.1 ± 0.13	0.0028 ± 0.0012	0.13 ± 0.095
Micro-plasmin (30 nM)	Boc-EKK-AMC	290 ± 74	3.1 ± 0.21	0.011 ± 0.0028	0.51 ± 0.33

Michaelis–Menten kinetics parameters and relative catalytic efficiencies of plasmin variants toward FPS or Boc-Glu-Lys-Lys-AMC. The means with SEM (*n* = 3) are shown.

FPS, fluorescence resonance energy quenching–based plasmin sensor.

To determine the mechanism of how plasmin’s kringle domains interact with FPS, we compared the initial rates of hydrolysis of FPS by mini-plasmin and plasmin in the presence and absence of inhibitory concentrations of TXA. The rate of cleavage of FPS by mini-plasmin was not altered by addition of TXA (Supplementary Figure 3). In contrast, TXA increased the rate of plasmin cleavage of FPS by 2.2-fold, making it similar to the rate of FPS cleavage by mini-plasmin (Supplementary Figure 3). Therefore, TXA does not alter FPS engagement with plasmin’s catalytic domain and kringle domain 5 but alters interactions between FPS and plasmin’s kringle domains 1 to 4.

### 3.3 FPS is as specific for plasmin as Boc-Glu-Lys-Lys-AMC

To compare the specificity of FPS and Boc-Glu-Lys-Lys-AMC for plasmin, we examined the rates of their hydrolysis by various coagulation and fibrinolytic proteases in buffer. Compared with plasmin, kallikrein cleaved FPS at 5.2% and Boc-Glu-Lys-Lys-AMC at 4.3%; FXIa cleaved FPS at 2.7%; and Boc-Glu-Lys-Lys-AMC at 1.4% (Table 2). The remaining proteases did not detectably cleave FPS or Boc-Glu-Lys-Lys-AMC (Table 2). These data suggest that the specificity of FPS and Boc-Glu-Lys-Lys-AMC for plasmin are comparable [1].

**Table 2:** The rates of cleavage of FPS and Boc-Glu-Lys-Lys-AMC relative to plasmin.

Enzyme	FPS	Boc-EKK-AMC
Plasmin	1.0 ± 0.043	1.0 ± 0.013
Thrombin	<0.01	<0.01
aPC	<0.01	<0.01
Factor Xa	<0.01	<0.01
tPA	<0.01	<0.01
uPA	<0.01	<0.01
Factor IXa	<0.01	<0.01
Factor XIa	0.027 ± 0.0029	0.014 ± 0.00016
Kallikrein	0.052 ± 0.0020	0.043 ± 0.00042
Plasmin with α_2_-M	<0.01	0.51 ± 0.0058

The cleavage rates of FPS and Boc-Glu-Lys-Lys-AMC by plasmin compared with other proteases. The means and SEM (*n* = 3) are shown. For the α_2_-M experiment, α_2_-M was preincubated with plasmin for 1 hour to produce α_2_-M plasmin complexes prior to addition of the substrates.

α_2_-M, α_2_-macroglobulin; aPC, activated protein C; FPS, fluorescence resonance energy quenching–based plasmin sensor; tPA, tissue-type plasminogen activator; uPA, urokinase-type plasminogen activator.

### 3.4 FPS is insensitive to the α_2_-M–plasmin complex

We examined whether the FPS could be cleaved by plasmin entrapped by α_2_-M. Plasmin preincubated with α_2_-M exhibited a 49% reduction in relative rates compared with Boc-Glu-Lys-Lys-AMC (Table 2). In contrast, FPS was not cleaved by plasmin preincubated with α_2_-M at the same concentration (Table 2). Therefore, unlike Boc-Glu-Lys-Lys-AMC, FPS is not cleaved by entrapped plasmin.

### 3.5 FPS measures plasmin generation in plasma

To determine the appropriate concentration of FPS to monitor tPA-initiated plasmin generation in plasma clot lysis assays, we assessed the saturating concentration of FPS in these assays and evaluated whether increasing concentrations of FPS impacted clot lysis. EPP and peak heights were not significantly different between assays measured using 0.25 to 1 μM of FPS (Supplementary Figure 4A, B). However, peak times were significantly shorter with 0.25 μM FPS than those with 0.5 μM FPS, but there was no difference between 0.5 and 1 μM FPS (Supplementary Figure 4C). These results indicate that FPS concentrations >0.5 μM are saturating and thus sufficient to monitor plasmin generation in plasma clot lysis assays (Supplementary Figure 4). Furthermore, when clot formation and lysis were monitored by light absorption, no significant changes in maximum absorbance and half clot lysis times were observed across these concentrations of FPS (Supplementary Figure 5). FPS at 2 μM inhibited clot formation (data not shown). Taken together, these data suggest that FPS up to 1 μM does not alter plasma clot formation and lysis under these experimental conditions.

### 3.6 FPS measures the effects of plasmin generation inhibition by AP and aprotinin

**Figure 1:**
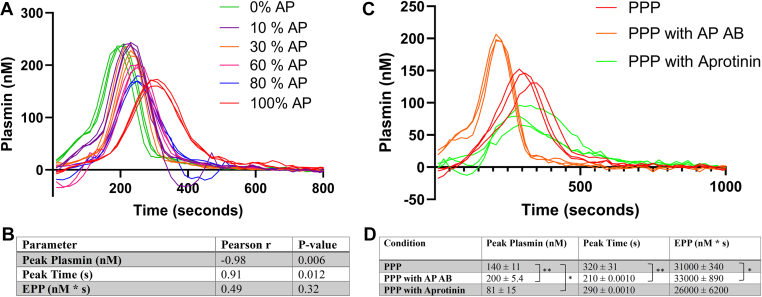
Fluorescence resonance energy quenching–based plasmin sensor measures plasmin generation in plasma, effect of α_2_-AP inhibition and aprotinin. (A) Plasmin generation profiles (*n* = 3) for when normal pooled platelet-poor plasma (PPP) was mixed with various amounts of α_2_-antiplasmin (AP)–deficient plasma are shown. The labels indicate the percentage of normal PPP, thus the percentage of AP. (B) Pearson correlation coefficients, with *P* values for the percentage of AP and parameters of the plasmin generation curves in (A) are shown. (C) Plasmin generation profiles (*n* = 3) comparing the presence (orange) and absence (red) of 0.5 μM antibody against AP (AP AB) and the presence (green) of 1 μM aprotinin added to PPP are shown. (D) The means with SDs for the peak plasmin, peak time, and endogenous plasmin potential (EPP) for the plasmin generation curves in (C) with significant differences between the groups are shown. ∗*P* < .05; ∗∗*P* < .01.

To determine the sensitivity of the FPS to plasmin inhibition, we performed plasmin generation assays using FPS with plasma containing (1) various percentages of residual AP activity generated by mixing PPP with AP-deficient plasma, (2) AP AB, or (3) aprotinin. Increased levels of AP activity in plasma were associated with decreased peak heights (*r* = −0.98) and increased time-to-peak (*r* = 0.91) plasmin generation in a dose-dependent manner (Figure 1A, B). The half-maximal peak height and peak time were 200 ± 5.6 nM plasmin and 250 ± 12 seconds, respectively, corresponding to ∼60% AP. Plasmin generation with the addition of AP AB to normal PPP was like that in AP-deficient plasma (Figure 1). Consequently, AP AB was used to simulate AP-deficient plasma for all subsequent experiments. The addition of aprotinin resulted in a decrease in peak plasmin. However, plasmin generation was still detected in the presence of aprotinin (Figure 1C, D). These results suggest that FPS can measure the inhibitory effects of AP and aprotinin.

### 3.7 FPS measures the effects of TXA

**Figure 2:**
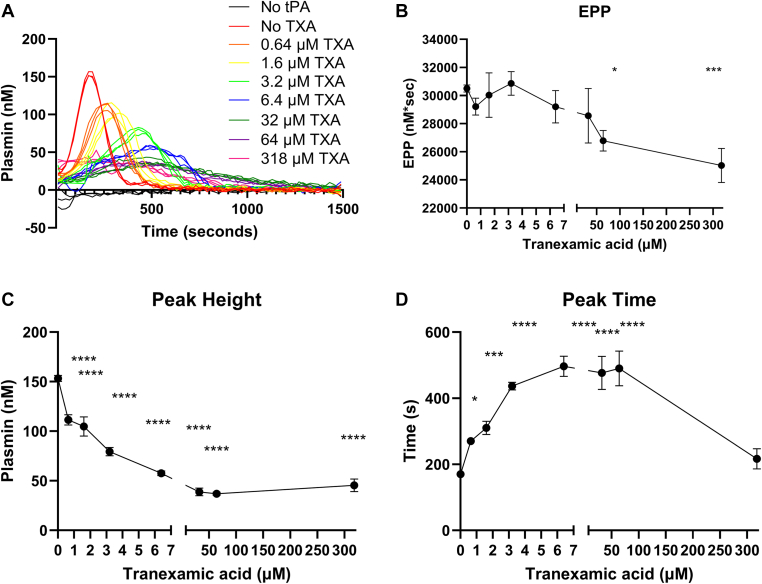
Fluorescence resonance energy quenching–based plasmin sensor (FPS) measures plasmin generation in plasma, effect of tranexamic acid (TXA). (A) Plasmin generation profiles (*n* = 3) for when 0.64 to 318 μM TXA were added to normal pooled platelet-poor plasma with the FPS along with a no–tissue-type plasminogen activator negative control are shown. The means with SDs for the endogenous plasmin potential (EPP) (B), peak plasmin (C), and peak time (D) for the plasmin generation curves in (A) plotted against the concentration of TXA with significant differences between the tested TXA concentration and no TXA group are shown. ∗*P* < .05; ∗∗∗*P* < .001; ∗∗∗∗*P* < .0001.

FPS measured plasmin generation can detect TXA-mediated inhibition of plasmin generation at TXA concentrations down to 0.64 μM in a dose-dependent manner (Figure 2). When no tPA was added to the plasma with thrombin and FPS, no FPS cleavage was detected (Figure 2A).

### 3.8 Evaluating the effects of fibrin on plasmin generation using FPS

**Figure 3:**
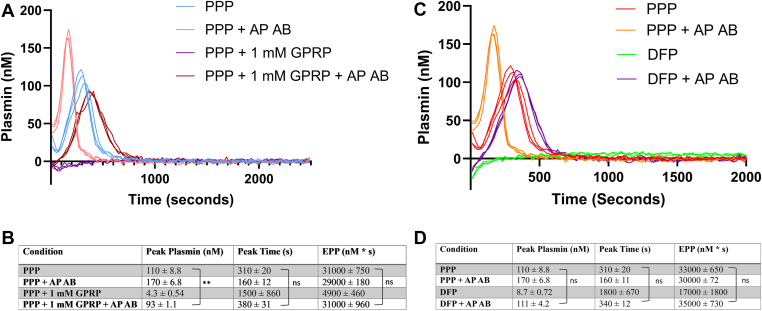
Fluorescence resonance energy quenching–based plasmin sensor (FPS) measures plasmin generation in plasma, effect of fibrin. (A) Plasmin generation profiles in normal pooled platelet-poor plasma (PPP; *n* = 3) for when 1 mM (purple) Gly-Pro-Arg-Pro (GPRP) were added with the FPS are shown. This experiment was also done in the presence (pink) and absence (light blue) of 0.5 μM antibody against antiplasmin (AP AB). Simultaneous addition of 0.5 μM AP AB with 1 mM (brown) GPRP was also evaluated. (B) The means with SDs for the peak plasmin, peak time, and endogenous plasmin potential (EPP) for the plasmin generation curves in (A) are shown. Peak plasmin was significantly higher in plasma with no GPRP compared with plasma with simultaneous addition of GPRP and AP AB (∗∗*P* < .01). (C) Plasmin generation profiles (*n* = 3) comparing PPP (red) and defibrinated plasma (DFP; green) are shown. This experiment was also repeated for PPP (orange) and DFP (blue) in the presence of 0.5 μM AP AB. (D) The means with SDs for the peak plasmin, peak time, and EPP for the plasmin generation curves in (C) are shown, where no significant (ns; *P* > .05) differences in plasmin generation parameters were observed between PPP and DFP with the addition of AP AB.

Fibrin fibrils and polymerized fibrin are cofactors for plasmin generation. The addition of 1 mM GPRP, an inhibitor of fibrin polymerization, to a plasmin generation assay, measured using FPS, nearly abolished plasmin generation (Figure 3A, B). Simultaneous addition of AP AB with 1 mM GPRP restored plasmin generation to be similar to normal PPP, where EPP and peak times were not significantly different (Figure 3A, B).

Plasmin generation, measured using FPS, was barely observed in defibrinated plasma where no fibrin fibrils would be present (Figure 3C, D). However, the addition of AP AB restored plasmin generation to that in normal PPP, where EPP, peak times, and peak heights were not significantly different. When no thrombin was added to initiate clotting in normal pooled plasma, results were similar to defibrinated plasma in which minimal plasmin generation was observed (data not shown).

To determine the basis of this result with AP AB, we determined the minimum threshold for detecting plasmin activity using FPS in normal pooled plasma in the presence and absence of AP AB. The minimum plasmin concentration required to obtain detectable plasmin activity in the absence of AP AB was 75 nM (data not shown); however, when AP AB was added, 7 nM plasmin was the minimum concentration required to cleave FPS (data not shown). These results indicated that, with the AP AB, FPS can measure the small amount of plasmin generated in this assay in the absence of fibrin fibrils, but more plasmin is generated when fibrils are present.

### 3.9 FPS allows novel evaluation of the plasminogen–tPA–fibrin interaction

**Figure 4:**
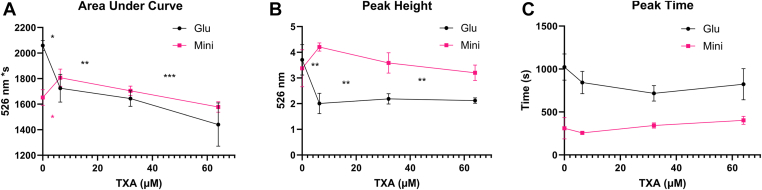
The interaction between plasmin’s kringle domain 5 and the tissue-type plasminogen activator–fibrin complex. The means with SDs for the area under the curve (A), peak height (B), and peak time (C) of tissue-type plasminogen activator mediated mini-plasminogen (Mini) and Glu-plasminogen (Glu) activation experiments (*n* = 3) measured using the fluorescence resonance energy quenching–based plasmin sensor in the presence of 0 to 64 μM tranexamic acid (TXA) are shown. Significant decreases in area under the curve and peak height were observed for Glu-plasminogen in a TXA dose-dependent manner, while 6.4 μM TXA significantly increased the area under the curve compared with the control for mini-plasminogen (∗*P* < .05; ∗∗*P* < .01; ∗∗∗*P* < .001). However, TXA did not significantly alter the peak times for both Glu-plasminogen and mini-plasminogen (nonsignificant [ns]; *P* > .05).

Kringle 5 has been reported to interact with a cryptic site on tPA in the tPA–fibrin complex [13]. It is unknown whether this is lysine dependent [13]. Because of the unique dependence of FPS on kringle 5, we measured the effect of TXA on tPA-mediated activation of Glu-plasminogen and mini-plasminogen with FPS plasmin generation to better understand this mechanism [13]. Glu-plasminogen activation was sensitive to the inhibitory effects of TXA since all tested concentrations of TXA significantly reduced the peak height and area under the curve (Figure 4A, B). In contrast, TXA increased mini-plasminogen activation since 6.4 μM TXA resulted in a greater area under the curve than that measured in its absence (Figure 4A).

### 3.10 FPS detects PAI-1 effects on plasma plasmin generation

**Figure 5:**
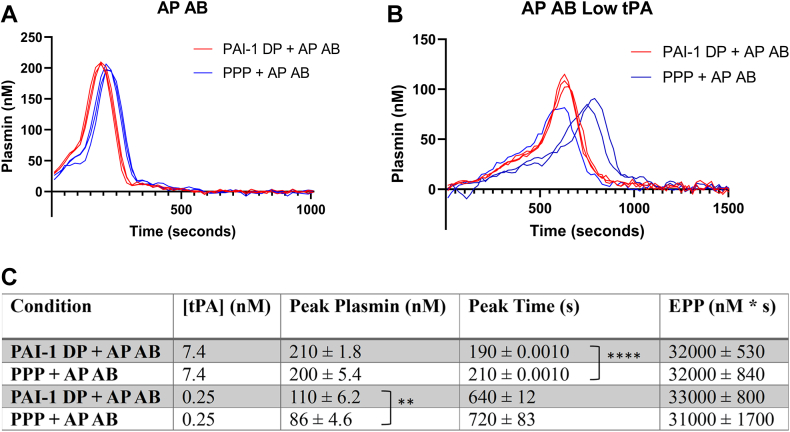
Fluorescence resonance energy quenching–based plasmin sensor (FPS) detects the effects of plasminogen activator inhibitor (PAI)-1 in plasma. (A) Plasmin generation profiles (*n* = 3) in normal pooled platelet-poor plasma (PPP; blue) and in PAI-1–deficient plasma (DP; red) with the addition of 0.5 μM antibody against antiplasmin (AP AB) measured using the FPS where plasmin generation was initiated with 7.4 nM tissue-type plasminogen activator (tPA) are shown. (B) Plasmin generation profiles (*n* = 3) for the experiments in (A) repeated using 0.25 nM tPA are shown. (C) The means with SDs for the peak plasmin, peak time, and endogenous plasmin potential (EPP) for the plasmin generation curves in (A) and (B) are shown. The peak times were significantly different between PAI-1 DP and normal pooled plasma in the presence of AP AB (∗∗∗∗*P* < .0001) when 7.4 nM tPA was used, while the peak heights were significantly different between PAI-1 DP and normal pooled plasma (∗∗*P* < .01) when 0.25 nM tPA was used.

PAI-1 has a short active half-life *in vivo* and in stored plasma [17]. We evaluated whether the FPS could detect the effects of PAI-1. To do this, we compared the plasmin generation profiles in normal and PAI-1–deficient plasma. Plasmin generation parameters were not significantly different between control and PAI-1–deficient plasma (Supplementary Figure 6). However, when AP AB was added, the plasmin peak time was shorter in PAI-1–deficient plasma than in control plasma (Figure 5A, C). These results indicate that FPS can measure PAI-1 activity when AP is inhibited.

We then explored whether using a lower tPA concentration to induce plasmin generation in combination with AP AB can lead to greater PAI-1 sensitivity. With 0.25 nM tPA, the peak height was lower in control plasma than in PAI-1–deficient plasma (Figure 5B, C). However, EPP and peak times were not different between the plasma samples (Figure 5B, C).

### 3.11 Comparing plasmin generation with clot lysis when plasmin generation is measured using FPS and Boc-Glu-Lys-Lys-AMC

**Figure 6:**
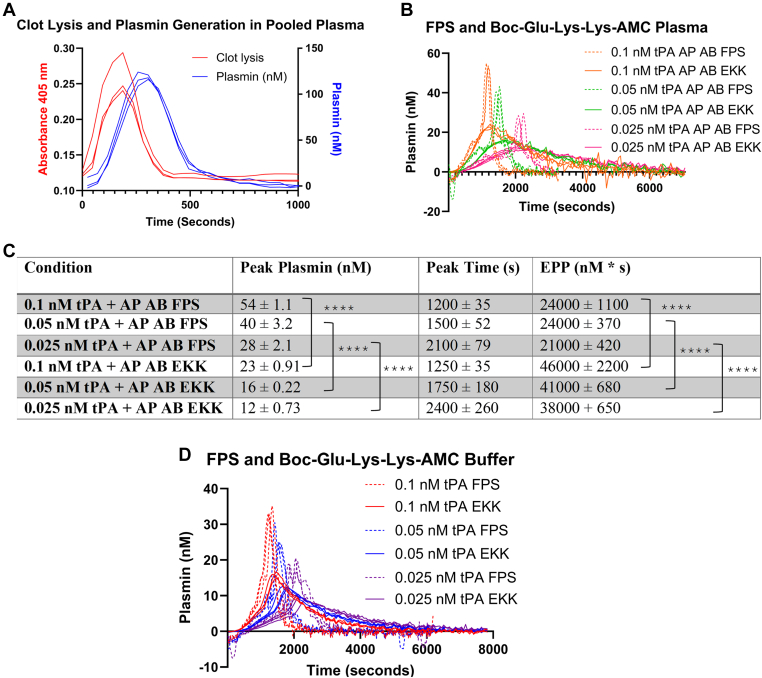

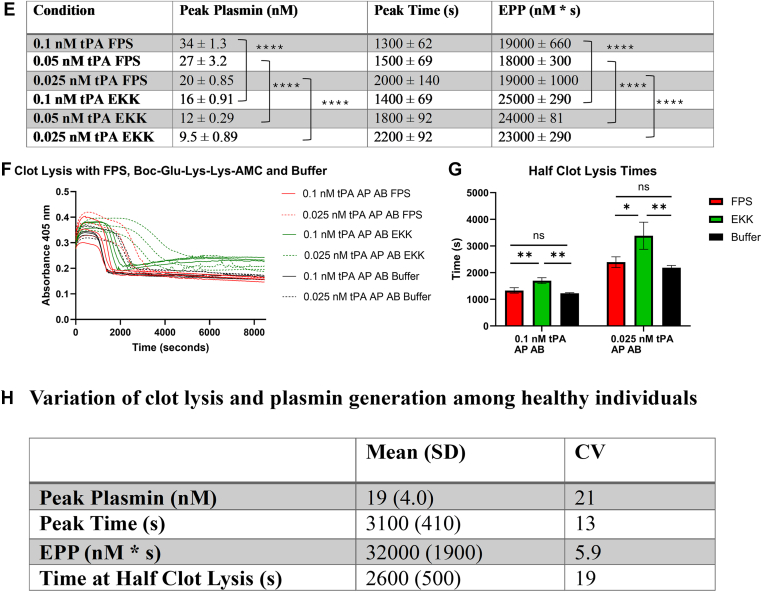
Plasmin generation and clot lysis measured using fluorescence resonance energy quenching–based plasmin sensor (FPS) and Boc-Glu-Lys-Lys-AMC. (A) Plasmin generation profiles and clot lysis profiles (*n* = 3) in normal pooled plasma obtained by simultaneously measuring FPS hydrolysis and turbidity are shown. These profiles were measured using 1 μM of the FPS where plasmin generation was initiated using 7.4 nM tissue-type plasminogen activator (tPA). (B) Plasmin generation profiles in normal pooled platelet-poor plasma (*n* = 3) measured using 1 μM FPS (dashed line) and 1 mM Boc-Glu-Lys-Lys-AMC (EKK; solid line) are shown. For these experiments, 0.1 (orange), 0.05 (green), and 0.025 (pink) nM tPA were used to initiate plasmin generation in the presence of 0.5 μM antibody against antiplasmin (AP AB). (C) The means with SDs for the peak plasmin, peak time, and endogenous plasmin potential (EPP) for the plasmin generation curves in (B) are shown. For all tested tPA concentrations, peak plasmin and EPP were significantly different between experiments measured using FPS and EKK (∗∗∗∗*P* < .0001). (D) Plasmin generation profiles (*n* = 3) in buffer with 1 μM fibrinogen and 50 nM Glu-plasminogen measured using 1 μM FPS (dashed line) and 1 mM EKK (solid line) where plasmin generation was initiated using 0.1 (red), 0.05 (blue), and 0.025 (purple) nM tPA are shown. (E) The means with SDs for the peak plasmin, peak time, and EPP for the plasmin generation curves in (D) are shown. For all tested tPA concentrations, peak plasmin and EPP were significantly different between experiments measured using FPS and EKK (∗∗∗∗*P* < .0001). (F) Clot lysis profiles (*n* = 3) in normal pooled plasma measured with the addition of 1 μM FPS (orange), 1 mM EKK (green), or Tris-buffered saline (black) in the presence of 0.5 μM AP AB are shown. For these experiments, 0.1 (solid line) and 0.025 (dashed line) nM tPA were used to initiate plasmin generation. (G) The half clot lysis times for when 0.1 and 0.025 nM tPA were used to initiate plasmin generation in the presence of 0.5 μM AP AB in (F) are shown. For these experiments, there were no significant differences in half clot lysis times between when buffer and FPS were added to plasma (nonsignificant [ns]; *P* > .05). However, EKK significantly altered half clot lysis times when compared with buffer (∗∗*P* < .01). (H) The means with SDs and coefficient of variations (CVs) for the peak plasmin, peak time, EPP, and half clot lysis times for plasmin generation profiles and clot lysis profiles of 13 individuals (*n* = 3 for 12 individuals; *n* = 2 for 1 individual because of experimental error) when 0.5 nM tPA was used to initiate plasmin generation are shown.

Since plasmin is the main effector of fibrinolysis, we determined how plasmin activity correlated with clot lysis [18]. This was performed by measuring plasmin generation using the FPS while simultaneously measuring clot formation and lysis by light absorption to assess clot turbidity. Plasmin generation in pooled PPP occurs along with clot formation, and peak plasmin occurs at the time of 50% clot lysis (Figure 6A). This pattern also occurred with a lower tPA concentration where the plasmin peak time was longer (data not shown), thus allowing more time for the clot to form before lysis.

We compared plasma plasmin generation measured using FPS with plasma plasmin generation measured using Boc-Glu-Lys-Lys-AMC in the presence of AP AB. For all tested tPA concentrations (0.025-0.1 nM), FPS measured more than 2-fold higher peak plasmin and close to 2-fold lower EPP than Boc-Glu-Lys-Lys-AMC; however, peak times were not significantly different (Figure 6B, C). To understand this disparity better, we evaluated plasmin generation in an isolated system where α_2_-M was not present. Thus, in a buffer system with fibrinogen and Glu-plasminogen, with fibrin formation initiated by thrombin and lysis initiated by tPA, we compared plasmin generation parameters in assays using either FPS or Boc-Glu-Lys-Lys-AMC. We observed a similar pattern where, compared with using FPS, Boc-Glu-Lys-Lys-AMC measured significantly higher EPP, >2-fold lower peak plasmin while having the same peak times (Figure 6D, E). These results suggested that the differences in plasmin generation parameters between FPS and Boc-Glu-Lys-Lys-AMC in plasma could not be fully explained by α_2_-M.

To further evaluate the difference in plasmin generation observed between FPS and Boc-Glu-Lys-Lys-AMC, we measured the clot lysis profiles of plasma containing AP AB and with FPS, Boc-Glu-Lys-Lys-AMC, or buffer. Plasma samples containing Boc-Glu-Lys-Lys-AMC had significantly higher half clot lysis times than samples with buffer added, whereas samples containing FPS were not significantly different from those where buffer was added; this occurred when either 0.025 or 0.1 nM tPA were used to initiate plasmin generation (Figure 6F, G). These results demonstrate that Boc-Glu-Lys-Lys-AMC alters clot lysis whereas FPS does not.

To investigate the variability in tPA-initiated plasmin generation and consequent clot lysis, we measured them simultaneously in plasma from 13 healthy donors (6 males and 7 females). No significant differences in lysis times and plasmin generation parameters were found between male and female donors; thus, data for all individuals were analyzed together. As expected, lysis times directly correlated with peak times (*r* = 0.77) and inversely correlated with peak heights (*r* = −0.55) and EPP (*r* = −0.38). The coefficients of variation for all individuals for each parameter were as follows: peak plasmin, 21%; peak time, 13%; EPP, 5.9%; and half clot lysis time, 19% (Figure 6H); the range from minimum to maximum was <2-fold.

## 4 Discussion

In this study, we report the synthesis of a macromolecular plasmin substrate (FPS) that is at least 10-fold more efficiently cleaved by plasmin than small peptide substrates, interacts with plasmin’s kringle domain 5, and is not cleaved by plasmin bound to α_2_-M [2,19]. Similar to our previously reported thrombin-specific substrate, a probable explanation for the FPS being resistant to cleavage by α_2_-M–bound plasmin is that FPS is a large protein, which hinders its access to plasmin’s active site when entrapped within α_2_-M [10,19]. In addition, with an inhibitory antibody to AP, we are able to detect effects of PAI-1 on plasmin generation. Lastly, we report that the variability of plasmin generation parameters using the FPS among healthy individuals is low and comparable with simultaneous clot lysis times.

Currently, there are no assays that can reliably quantify real-time plasmin activity in plasma [1,7,[20], [21], [22], [23]]. The major limitation of using small-molecule substrates such as Boc-Glu-Lys-Lys-AMC and Cbz-Phe-Arg-rhodamine-morpholino to measure plasmin generation is that they are able to access plasmin’s cleavage site and be cleaved by plasmin encapsulated by α_2_-M [19]. Other types of assays that detect plasmin activity using small peptide substrates include electrochemical and acoustic methods, which are also sensitive to α_2_-M–entrapped plasmin [20,21]. This is a challenge because plasmin that is in complex with α_2_-M cannot lyse fibrin, thus leading to an overestimation of total plasmin potential in a plasma sample [24]. A correction assuming equal α_2_-M concentration in plasma ignores the reported variation of α_2_-M concentration in health and disease [25]. For example, patients with liver cirrhosis have higher α_2_-M levels than healthy individuals [25]. Furthermore, newborns and young children have 2- to 3-fold higher α_2_-M levels relative to adults [26]. Although methods such as ELISAs and a bioluminescence resonance energy transfer plasmin sensor are insensitive to α_2_-M–inhibited plasmin, these methods are unable to measure real-time plasmin activity [22,23]. Thus, there is an unmet need for a reliable method of measuring plasmin activity that will permit a comprehensive understanding of plasmin generation during clinical settings.

Plasmin’s specificity toward its substrates is determined by active site features and its kringle domains that bind to exposed lysine residues [2,11]. The plasmin recognition sequence (GVYKSRSL) of FPS is based on the previously established optimal plasmin recognition sequence found by phage display and the sequence of the whole FPS contains 56 lysine residues that may participate in plasmin binding [11,12]. The low *K*_m_ of the FPS for plasmin leads to it having a 9-fold higher catalytic efficiency for plasmin than Boc-Glu-Lys-Lys-AMC (Supplementary Table). Furthermore, the FPS is 23-fold more efficiently cleaved by plasmin relative to published results for Cbz-Phe-Arg-rhodamine-morpholino (0.0077 μM^−1^ s^−1^) (Supplementary Table) [7]. Moreover, the FPS is 3.6-fold more efficiently cleaved by plasmin than S-2251 (0.05 μM^−1^ s^−1^) (Supplementary Table) [27]. This suggests that the ability of the FPS to accommodate a long plasmin recognition sequence and/or form kringle domain interactions with plasmin might contribute to its high efficiency compared with small peptide substrates.

Our results show that cleavage of FPS by plasmin is enhanced by interactions between FPS and plasmin’s kringle domain 5 and suppressed by interactions between FPS and plasmin’s kringle domains 1 to 4. This is evident since mini-plasmin, which has only kringle domain 5, cleaved FPS with similar efficiency to plasmin. In addition, the catalytic efficiency of micro-plasmin, which has no kringle domains, for FPS is reduced compared with that of plasmin, reflecting a lower *k*_cat_. These findings suggest that the presence of plasmin’s kringle 5 enhances the ability of plasmin’s active site to cleave FPS; this implies an allosteric interaction between kringle 5 and the active site. Furthermore, interactions between FPS and kringle 5 appear to be lysine independent, whereas FPS interacts with kringles 1 to 4 in a lysine-dependent manner, since our data showed that TXA enhanced plasmin’s ability to cleave FPS but had no effect on mini-plasmin’s ability to cleave FPS. These results are also consistent with our finding that mini-plasmin has not only a higher *k*_cat_ but also a higher *K*_m_ than plasmin for FPS, which resulted in it having the same efficiency as plasmin. Since mini-plasmin only has kringle 5, it binds FPS with lower affinity than plasmin, which gives mini-plasmin a higher *K*_m_. However, kringles 1 to 4 suppresses plasmin’s ability to cleave FPS, which allows mini-plasmin to have a higher *k*_cat_ than plasmin. The importance of kringle 5 for FPS cleavage is also emphasized by the contrast between it and Boc-Glu-Lys-Lys-AMC when comparing catalytic efficiencies between mini-plasmin and micro-plasmin, whereby the addition of kringle 5 to the catalytic domain increases the efficiency of FPS by 13-fold, while adding kringle 5 to the catalytic domain decreases the efficiency of Boc-Glu-Lys-Lys-AMC by 4-fold.

The ability of the FPS to interact with kringle domain 5 of plasmin/plasminogen adds insight to the mechanism by which plasminogen interacts with the tPA–fibrin complex. Unlike kringle domains 1 to 4 of plasminogen, which bind to fibrin, kringle 5 was previously shown to either bind fibrin or bind to a cryptic site on tPA during tPA-mediated plasminogen activation [13]. Our results show that TXA addition decreased the peak height and area under the curves of Glu-plasminogen activation by tPA but mini-plasminogen activation was increased by TXA addition. Furthermore, our data show that when TXA is added plasmin and mini-plasmin cleave FPS equally well. Thus, the inhibitory effects of TXA on tPA mediated Glu-plasminogen activation are a result of plasminogen activation rather than plasmin activity and overall, our data support kringle 5 binding to tPA rather than to fibrin [13].

FPS at the concentration used in plasma assays (1 μM) did not impact clot formation and lysis when fibrinolysis was initiated with tPA. This suggests that FPS at experimentally desirable concentrations does not interfere with plasminogen activation or plasmin activity. Thus, although FPS can specifically interact with kringle 5 of plasmin, which is essential for activation of plasminogen by tPA when fibrin is present, FPS does not inhibit plasmin or plasminogen activation [13]. A possible explanation could be that the off-rate: on-rate ratio of plasmin for FPS is relatively high compared with tPA for plasminogen and plasmin for its substrates, which would lead to interactions between plasmin and FPS being too transient to affect plasminogen activation and plasmin activity [28]. However, a limitation of FPS is that it inhibits clotting when used at concentrations >2 μM. This suggests that the use of FPS might be limited to relatively optically transparent systems such as plasma, where the use of high concentrations of FPS is not required.

When compared with FPS, Boc-Glu-Lys-Lys-AMC has low efficiency for plasmin; thus, it has to be used in the mM range in plasma assays [24]. When Boc-Glu-Lys-Lys-AMC is cleaved by plasmin, it becomes Boc-Glu-Lys-Lys and Boc-Glu-Lys with carboxy-terminal lysine residues. Thus, adding high concentrations of Boc-Glu-Lys-Lys-AMC to plasma is essentially the same as adding lysine analogs such as TXA, and this effect will become more prominent as plasmin is generated to cleave Boc-Glu-Lys-Lys-AMC, which produces more free lysines [24]. Boc-Glu-Lys-Lys-AMC increases half clot lysis times in plasma assays, whereas FPS does not. Similar to Boc-Glu-Lys-Lys-AMC, SN-5, which is another fluorogenic substrate for plasmin that produces D-Ala-Phe-Lys when cleaved by plasmin, also significantly increases half clot lysis times of plasma (Supplementary Figure 7). Furthermore, TXA and ε-aminocaproic acid enhance the stability of bovine plasmin by preventing autodegradation of its active fragment (Met343-Asn786) [29]. This suggests that Boc-Glu-Lys-Lys-AMC might also have stabilizing effects on plasmin, which enables it to measure higher EPPs than FPS in both plasma and buffer plasmin generation assays.

Fibrinolytic therapies that inhibit AP have been developed to increase endogenous thrombolysis in patients with deep vein thrombosis and pulmonary embolism [30]. We have tested the effect of AP AB, an antibody inhibitor of AP, on plasmin generation assay parameters measured using FPS. Both plasma dilution with AP-deficient plasma and addition of AP AB to PPP increase peak plasmin and shorten plasmin peak time. This contrasts with the findings with Boc-Glu-Lys-Lys-AMC where changing AP levels only resulted in changes in peak plasmin [24]. The difference likely results from Boc-Glu-Lys-Lys-AMC or its products, altering clot lysis, which complicates the effects of changing AP levels. This suggests that using Boc-Glu-Lys-Lys-AMC might have produced misleading results when it is used to measure plasmin generation [24,[31], [32], [33]].

Plasma plasmin generation assays have shown that the addition of aprotinin resulted in decreased peak plasmin levels, which is consistent with our finding that FPS is specific for plasmin. However, since aprotinin is a reversible competitive inhibitor of plasmin (*k*_1_ = 0.91 μM^−1^ s^−1^) that is only 5-fold more efficient than plasmin for FPS (0.18 μM^−1^ s^−1^), it possible that some plasmin could be measured by FPS before it is inhibited by aprotinin [34].

Plasmin generation assays using FPS show that FPS was sensitive to TXA inhibition at 0.64 μM, which contrasts with Boc-Glu-Lys-Lys-AMC, which had a detection limit of 9.5 μM TXA [24]. Thus, FPS might provide an advantage over Boc-Glu-Lys-Lys-AMC for measuring the clearance of TXA if there is a need to measure low levels of TXA [35]. However, another method with a similar detection limit but using binding affinity rather than enzymatic activity has recently been published [36]. Given that the effective therapeutic plasma concentration of TXA for inhibiting fibrinolysis is 64 to 96 μM, FPS would be able to measure TXA levels in plasma in clinical settings [37]. Interestingly, our results show that TXA at 318 μM did not completely abolish plasmin generation, it, instead, made the plasmin generation curve lower and prolonged. Although this is consistent with results where plasmin generation was measured using Boc-Glu-Lys-Lys-AMC, where addition of 313 μM TXA also resulted in a low and prolonged plasmin generation curve [24]. However, given that Boc-Glu-Lys-Lys-AMC itself or its products altering clot lysis in a manner similar to lysine analogs, it shows that even higher concentrations of TXA were not able to abolish plasmin generation [24]. This again might be resulting from TXA’s ability to prevent plasmin autodegradation and protect plasmin from inhibition by AP at high concentrations [29,38].

It was previously known that fibrin clots protect plasmin from inhibition by AP, which allows more active plasmin to cleave its substrates; furthermore, fibrin monomers are cofactors for plasmin generation [5,39]. As expected, plasmin generation measured using FPS indicated that the absence of fibrinogen or inhibition of fibrin polymerization resulted in nearly absent plasmin generation, which is consistent with what has been reported with Boc-Glu-Lys-Lys-AMC and Cbz-Phe-Arg-rhodamine-morpholino [7,8]. However, addition of the AP AB antibody, which increased sensitivity to detect plasmin by 10-fold, resulted in measurable plasmin activity with FPS in the absence of fibrin/fibrinogen and in the absence of polymerization.

Moreover, our data indicate that only when AP is inhibited, increasing the sensitivity of plasmin detection, can plasmin generation measured using FPS distinguish between normal pooled plasma and PAI-1–deficient plasma. This contrasts with a report where plasmin generation parameters (peak height and EPP) are increased in PAI-1–deficient patients [6]. However, that report used Cbz-Phe-Arg-rhodamine-morpholino, a sensor that is cleaved by thrombin and FXa in addition to plasmin, suggesting that varied generation of these proteases may have contributed to the results in PAI-1–deficient patients [1,6]. However, our results are consistent with reports of Boc-Glu-Lys-Lys-AMC being insensitive to PAI-1, although not tested with AP inhibition [8]. PAI-1 antigen levels are ∼0.47 nM in normal pooled PPP [40]. However, PAI-1 is unstable with a half-life of 1 to 2 hours in the circulation [17]. Thus, plasma, which takes time to process, contains extremely low levels of active PAI-1 [17]. This suggests that when AP is inhibited, FPS is sensitive to the small differences between normal and PAI-1–deficient plasma in plasmin activity generated by added tPA.

Simultaneous clot lysis and plasmin generation assays in normal pooled plasma measured using FPS showed that peak plasmin occurs at the same time as 50% clot lysis; this observation was consistent over a range of tPA concentrations that resulted in peak plasmin times between 300 and 3000 seconds. This is consistent with purified systems where tPA and plasminogen were laid onto fibrin clots; maximum plasmin generation occurs approximately around or after half clot lysis [41]. Similarly, clot lysis occurred after peak plasmin with purified fibrin clots where fibrinolysis was initiated with tPA and plasminogen in the presence of AP in assays measured using S-2251 [39]. Moreover, maximal plasmin generation occurred around maximal clot lysis for plasma clots where plasminogen, which was premixed with casein, was activated by streptokinase and plasmin generation was determined by measuring acid-soluble tyrosine [42]. However, when tPA initiated plasma plasmin generation was measured using Boc-Glu-Lys-Lys-AMC, peak plasmin occurred at a similar time as maximum clotting has been reached [24]. In these experimental conditions, maximal clotting was at ∼300 seconds, which is likely too rapid to accurately assess plasmin generation with Boc-Glu-Lys-Lys-AMC [24]. Thus, in that report, when TXA was added to the same reaction, peak plasmin occurred after lysis began [24]. Furthermore, since Boc-Glu-Lys-Lys-AMC or its products inhibit clot lysis, this complicates interpretation of the plasmin generation profile. In our plasmin generation experiments with FPS, the tPA concentration directly correlated with peak plasmin, inversely correlated with peak time, but did not affect EPP (Supplementary Figure 8). This is mostly consistent with experiments using Boc-Glu-Lys-Lys-AMC where lower tPA resulted in lower peak plasmin and increased peak time [24].

Furthermore, our data have shown that variation of thrombin-induced plasmin generation parameters are comparable with half clot lysis times for healthy individuals, with a coefficient of variation of 5.9% to 21% and with a 2-fold difference between minimum and maximum. This contrasts with thrombin generation, which is highly variable with 4-fold differences in parameters in a healthy population [43]. The variation we report is lower than that reported for Boc-Glu-Lys-Lys-AMC in an assay where clotting is initiated by tissue factor [24]. Our results also differ from previous findings that showed that plasminogen activity varied from 25% to 200% across the population when plasminogen was activated using streptokinase and measured using S-2251 [44]. These might be a result from peptide substrates having the limitation of measuring α_2_-M–bound plasmin since α_2_-M levels vary by ∼5-fold in the healthy population [45]. Variation with S-2251 could also reflect different responses to plasminogen activation by streptokinase since I streptokinase can be cleaved by plasmin to smaller intermediates, thus complicating the reaction [46].

In conclusion, we have constructed FPS for measuring plasmin activity. FPS does not inhibit clot lysis, has high specificity and selectivity for plasmin, and uniquely resists plasmin complexed with α_2_-M. These properties make FPS an ideal candidate to evaluate fibrinolytic anomalies in laboratory and clinical settings [1]. FPS will allow for the measurement of more physiological plasmin generation and may be able to offer better prediction of bleeding or thrombosis associated with defects in the fibrinolytic cascade.

## Supplementary material

Supplementary Data

Supplementary Figures F1-F8 and Table S1
