# Supplementary material for: A novel biosensor for measuring plasmin activity
Source: Res Pract Thromb Haemost. 2026 May 2;10(4):106629. doi: 10.1016/j.rpth.2026.106629 (PMC13254883; doi:10.1016/j.rpth.2026.106629)
Supplement: Supplementary Figures F1-F8 and Table S1 [file mmc2.docx]

Supplemental Methods

To convert raw data to plasmin generation curves and obtain plasmin generation parameters.

See Supplemental Excel file with *worksheets*. Note worksheet names are in italics.

This explains the workflow to obtain the data in Figure 2, as an example, from raw data. To simplify understanding, only the data for no TXA, no tPA and 32 μM TXA are shown.

A) The raw data, which is emission at 526 nm (relative units) fluorescence, over time when FPS is excited at 406 nM are shown in *Raw Data.*

B) The raw fluorescence values were processed in GraphPad Prism by taking the first derivative (slope) with second order smoothing (4 neighbors). The results are in *Slope from prism*.

C) A standard curve to convert slope to nM plasmin is obtained in separate experiments. Varying concentrations of purified plasmin were used to cleave the same concentration of FPS, used in the experiments, in the presence of 5 nM thrombin, in α_2_-antiplasmin deficient plasma. The initial rate is measured to obtain a slope. Then these slopes are plotted against the concentration of plasmin. A slope of the best-fit line converts the data in *Slope from prism* to worksheet *Data in nM* by division by the slope of the standard curve (0.0536). This what is plotted in Figure 2a.

D) This data is then copied into GraphPad Prism and analyzed using the area under curve function using the default settings. The total area for each experiment is the EPP value; the peak Y for each experiment is the peak height; the peak X is the peak time. The summary data are shown in Figures 2b, c and d.

An identical workflow is used to obtain the data in Figures 1, 3, 5, and 6 and Supplement Fig 6.

**Supplementary Material**

Plasmin cleavage site (GVYKSRSL)

2X Histidine


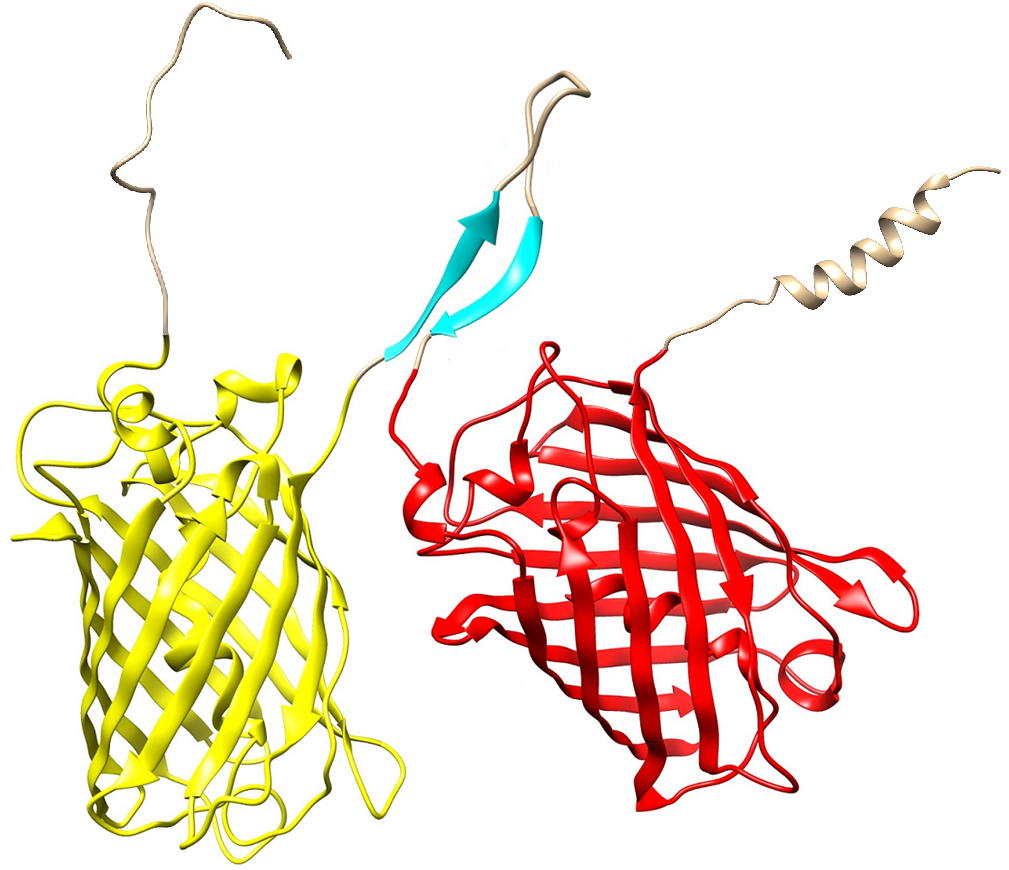


H beta from phototropin 2

I beta from phototropin 2

Calmodulin binding protein

tTomato

ex: 554 nm

em: 581 nm

mAmetrine

ex: 406 nm

em: 526 nm

**Supplemental Figure 1. A putative model of the FPS**. A possible ribbon structure of the FPS predicted using Phyre^2^ is shown with mAmetrine (yellow barrel) and tTomato (red barrel) linked by a plasmin recognition sequence (GVYKSRSL, beige loop). This recognition site is flanked by the H and I beta sheets of the light, oxygen, and voltage domain of phototropin 2 (blue sheets). The FPS is inserted between a 2X N-terminal 6X histidine tag (beige line) and a C-terminal calmodulin binding protein tag (beige helix). mAmetrine and tTomato were predicted to corresponded to green fluorescent protein (PDB ID: 4BDU) and red fluorescent protein (PDB ID: 1GGX), respectively. The plasmin recognition sequence and His-tags were predicted to form neither beta sheets nor helices while the CBP-tag was predicted to correspond to the myosin light chain kinase portion of calcium binding protein (PDB ID: 2BBM). The beta sheets were the H and I sheets from the LOV1 domain of phototropin 2 (PDB ID: 2Z6D), which were inserted into the model*. (Kelley, L. A., Mezulis, S., Yates, C. M., Wass, M. N. & Sternberg, M. J. E. The Phyre2 web portal for protein modeling, prediction and analysis. Nat. Protoc. 10, 845–858 (2015))*

(a)

MW Cells Supt CBP CBP Nickel Nickel

(kDa) FT Elute FT Eluate


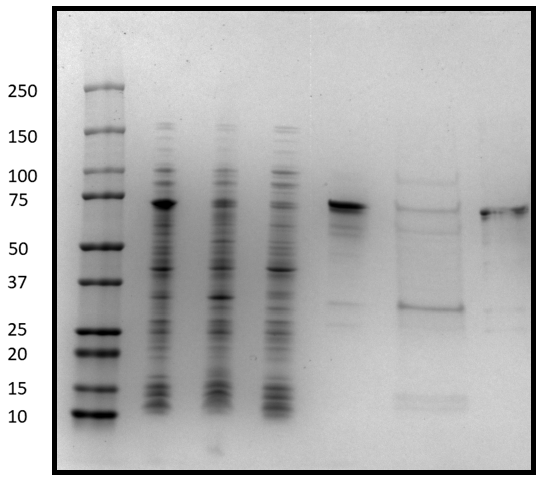


68 kDa

(b)

mAmetrine tTomato

Em: 526 nm Em: 581 nm

**Supplemental Figure 2. Purification and Spectra of the FPS.** (a) Samples from subsequent affinity-based FPS purification steps were resolved by SDS-PAGE (4%-15%) and visualized using Coomassie blue. Lanes containing the cells, soluble fraction of the *E.coli* lysate (Supt), calmodulin column flow through (CBP FT), calmodulin column elution (CBP Eluate) CBP Eluate that was subsequently flowed through a nickel column (Nickel FT) and the final nickel column elution (Nickel Eluate) are shown. The band corresponding to the size of the FPS is indicated by the arrow. (b) The emission spectra of the FPS before (blue) and after (purple) proteolysis by 100 nM plasmin for 1 hour when excited at 406 nm and measured at emission wavelengths from 500 to 640 nm are shown.

**Supplemental Figure 3. The effects of tranexamic acid on cleavage of the FPS by mini-plasmin and plasmin.** The means with standard deviations (N=3) for the rates of cleavage of 1 µM of FPS by 10 nM mini-plasmin (Mini) or plasmin in the presence and absence of 20 µM tranexamic acid (TXA) are shown. Addition of 20 µM TXA did not significantly impact (ns) cleavage of FPS by mini-plasmin but significantly increased plasmin mediated increase in 526 nM emission (^****^p < 0.0001) to levels that are not significantly (^ns^p > 0.05) different from mini-plasmin.

(a)

(b)

(c)

**Supplemental Figure 4. Determining the saturating concentration of FPS to be used in plasma plasmin generation assays.** The endogenous plasmin potential (EPP) (a), peak height (b) and peak time (c) for 1/3.5 diluted PPP in the presence of 5 nM thrombin and 7.4 nM tissue-type plasminogen activator (tPA) are shown. The means with standard deviations were determined (N=3). Using 0.25 µM of the FPS resulted in a significantly shorter peak time compared to 0.5 µM of the FPS (**p < 0.01).

(a)

(b)

**Supplemental Figure 5. Measuring the effects of the FPS on plasma clotting and lysis.** The half clot lysis times (a) and maximum absorbance (b) in 1/3.5 diluted PPP in the presence of 5 nM thrombin and 7.4 nM tissue-type plasminogen activator (tPA) are shown. The means with standard deviations were determined (N=3). The slopes of the half clot lysis times and maximum absorbances for all tested concentrations of the FPS were not statistically significantly different from zero (p > 0.05).

(a)

(b)

| Condition | [tPA] (nM) | Peak Plasmin (nM) | Peak Time (s) | EPP (nM * sec) |
| --- | --- | --- | --- | --- |
| PAI-1 DP | 7.4 | 140 ± 9.9 | 280 ± 12 | 30000 ± 870 |
| PPP | 7.4 | 143 ± 11 | 320 ± 31 | 31000 ± 330 |

**Supplemental Figure 6. FPS does not detect the effects of PAI-1 in plasma in the absence of α_2_-AP inhibition**. (a) Plasmin generation profiles (N=3) in normal pooled platelet-poor plasma (PPP) (green) and in plasminogen activator inhibitor-1 (PAI-1) deficient plasma (PAI-1 DP) (orange) measured using the FPS where plasmin generation was initiated with 7.4 nM tissue-type plasminogen activator (tPA) are shown. (b) The means with standard deviations for the peak plasmin, peak time, and endogenous plasmin potential (EPP) for the plasmin generation curves in (a) are shown. There were no significant differences between PPP and PAI-1 DP (^ns^p > 0.05).

**Supplemental Table 1**

**Catalytic efficiency of FPS for plasmin relative to other plasmin substrates**

| Substrate | k_cat_/K_m_ (s^-1^ µM^-1^) | Reference |
| --- | --- | --- |
| FPS | 0.18 | Table 1 |
| Boc-EKK-AMC | 0.021 | Table 1 |
| Cbz-Phe-Arg-rhodamine-morpholino | 0.0077 | [^7^] |
| S-2251 | 0.05 | [^26^] |

Catalytic efficiencies of plasmin substrates for plasmin determined by our experiments and taken from references [^7^ and ^26^] are shown.

**Supplemental Figure 7. The effects of** **D-Ala-Phe-Lys-ANSNH-iC_4_H_9_.2HBr on plasma clot lysis.** The half clot lysis times (N=3) for where 0.1 nM tPA was used to initiate plasmin generation the presence of 0.5 µM AP AB in normal pooled plasma measured with the addition of TBS (black) or 1 mM D-Ala-Phe-Lys-ANSNH-iC_4_H_9_.2HBr (SN-5) (blue) are shown. For these experiments, SN-5 significantly altered half clot lysis times when compared to buffer (**p < 0.01).

(a)

(b)

(c)

**Supplemental Figure 8. The effect of tPA concentration on plasmin generation curve parameters.** The endogenous plasmin potential (EPP) (a), peak height (b) and peak time (c) for 1/3.5 diluted PPP (N=9) in the presence of 5 nM thrombin and 7.4 nM tissue-type plasminogen activator (tPA) are shown and in plasma of 13 individuals (N=3 for 12 individuals, N=2 for 1 individual because of experimental error) where 0.5 nM tPA was used to initiate plasmin generation are shown. The means with standard deviations were determined and significantly different peak heights and peak times between low and high tPA concentrations were observed (****p < 0.0001).
